# Ancient DNA and the rewriting of human history: be sparing with Occam’s razor

**DOI:** 10.1186/s13059-015-0866-z

**Published:** 2016-01-11

**Authors:** Marc Haber, Massimo Mezzavilla, Yali Xue, Chris Tyler-Smith

**Affiliations:** The Wellcome Trust Sanger Institute, Wellcome Genome Campus, Hinxton, Cambridgeshire CB10 1SA UK; Institute for Maternal and Child Health, IRCCS BurloGarofolo, University of Trieste, 34137 Trieste, Italy

## Abstract

Ancient DNA research is revealing a human history far more complex than that inferred from parsimonious models based on modern DNA. Here, we review some of the key events in the peopling of the world in the light of the findings of work on ancient DNA.

## Background

The human past on many timescales is of broad intrinsic interest, and genetics contributes to our understanding of it, as do paleontology, archaeology, linguistics and other disciplines. Geneticists have long studied present-day populations to glean information about their past, using models to infer past population events such as migrations or replacements, generally invoking Occam’s razor to favor the simplest model consistent with the data. But this is not the most straightforward approach to understanding such events: the obvious way to study any aspect of human genetic history is to analyze population samples from before, during and after the period of interest, and to simply catalogue the changes. Advances in ancient DNA (aDNA) technology are now beginning to make this more direct approach possible, facilitated by new sequencing technologies that are now capable of generating gigabases of data at moderate cost (Box 1). This abundance of data, combined with an understanding of the damage patterns indicative of authentic aDNA, greatly simplify the recognition and avoidance of the bugbear of the field: contamination.

Here, we review some of the key events in the peopling of the world in the light of recent aDNA findings, discussing new evidence for how migration, admixture and selection have shaped human populations.

## Origin and expansion of modern humans and admixture with archaic species

For decades, the theories about the origin of modern humans were summarized in two main competing models: multiregional evolution or recent replacement from Africa [1, 2]. Genetic studies beginning in the 1980s provided explicit support for a recent origin of modern humans in Africa around 200,000 years ago (ya) [3], followed by an expansion out of Africa around 50,000–60,000 ya and subsequent colonization of the rest of the world [4].

There are hundreds of research papers discussing the out-of-Africa migration using archaeological data, present-day human genetic data or even genetic data from the human microbiome. Most of this work refines the recent replacement model, including suggesting a time-frame for the expansion [5] as well as the number of waves and routes taken by humans in their exit from Africa [4]. A few early studies did propose admixture with archaic humans [6, 7], but alternative interpretations of their examples were usually possible [8]. A major revision of the replacement model was introduced as the result of aDNA research published in 2010, in which DNA was retrieved from three Neanderthal bones from the Vindija Cave in Croatia [9] and from a finger bone found in Denisova Cave in southern Siberia [10]. Analyses of DNA from the archaic humans showed strong evidence of a small amount of gene flow to modern humans, giving rise to a ‘leaky replacement’ model. The initial report was met with some criticism, suggesting that ancient population substructure could produce a genetic signal similar to the one interpreted as introgression from Neanderthals [11] (see Box 2 for more details on the D-statistics relevant to this discussion). However, several later studies using different statistics showed that ancient structure alone cannot explain the introgression signal [12, 13].

Neanderthal ancestry in all present-day non-Africans is estimated to be 1.5–2.1 % [14]. The broad geographical distribution, together with the size of the DNA segments contributed by Neanderthals, suggests that the gene flow most likely occurred at an early stage of the out-of-Africa expansion: around 47,000–65,000 ya [12], before the divergence of Eurasian groups from each other. Sequences from the genomes of ancient Eurasians show that they carried longer archaic segments that have been affected by less recombination than those in present-day humans, consistent with the ancient individuals being closer to the time of the admixture event with Neanderthals. For example, a genome sequence from Kostenki 14 who lived in Russia 38,700–36,200 ya had a segment of Neanderthal ancestry of ~3 Mb on chromosome 6 [15], whereas present-day humans carry, on average, introgressed haplotypes of ~57 kb in length [16]. The genome sequence of a 45,000-year-old modern human male named Ust’-Ishim (after the region in Siberia where he was discovered), shows genomic segments of Neanderthal ancestry that are ~1.8–4.2 times longer than those observed in present-day individuals, suggesting that the Neanderthal gene flow occurred 232–430 generations before Ust’-Ishim lived, or approximately 50,000–60,000 ya [17], narrowing the previous range. Moreover, the Neanderthal-derived DNA in all non-Africans is more closely related to a Neanderthal from the Caucasus than it is to either the Neanderthal from Siberia or the Neanderthal from Croatia [14], providing more evidence that archaic admixture occurred in West Asia early during modern humans’ exit from Africa. It remains unclear how frequent mixture between Neanderthals and modern humans was, or how many Neanderthal individuals contributed; however, a higher level of Neanderthal ancestry in East Asians than in Europeans has been proposed to result from a second pulse of Neanderthal gene flow into the ancestors of East Asians [18, 19]. DNA from a 37,000–42,000-year-old modern human from Romania (named Oase) had 6–9 % Neanderthal-derived alleles, including three large segments of Neanderthal ancestry of over 50 centimorgans in size, suggesting that Oase had a Neanderthal ancestor as a fourth-, fifth- or sixth-degree relative [20]. The Oase population appears not to have contributed substantially to later humans in Europe, but the Oase genome provides direct evidence that multiple mixture events between modern humans and Neanderthals have occurred.

Admixture with Denisovans also occurred, possibly in South-East Asia [21], and affected the ancestors of present-day populations in Oceania, introducing 4–6 % Denisovan ancestry (in addition to their Neanderthal ancestry) in today’s New Guineans, Aboriginal Australians and Bougainville Islanders. A low level (~0.2 %) of Denisovan ancestry is also found across Eastern Eurasia and in Native American populations [14], but it is unclear whether this originated via gene flow from the same mixture event or through a second one. Denisovans themselves appear to have received gene flow from other archaic humans. It has been estimated that at least 0.5 % of the Denisovan genome was contributed by Neanderthals and that 0.5–8 % comes from an unknown hominin who split from other hominins between 1.1 and 4 million ya [14]. This complexity in the history of the archaic humans is also evident in the analysis of the oldest hominin sequenced to date: a 400,000-year-old individual from Sima de los Huesos in northern Spain. Their mitochondrial genome revealed evidence of a common ancestor shared with Denisovans rather than with Neanderthals [22], a finding that is surprising both as the Sima de los Huesos individual lived outside the known Denisovan geographical range and as the fossils carry Neanderthal-derived features. Scenarios to explain these results include gene flow between the different archaic species and/or a structure in the common ancestral population leading to Neanderthals, Denisovans and other *Homo* species. Future findings will likely show that many of the assumptions reported here were simplified and that, even with aDNA, we still have to invoke Occam’s razor to explain the data: that is, until sufficient human fossils have been sequenced.

aDNA evidence has thus supported the replacement model as an explanation for most human variation, but has transformed and enriched this model in ways not anticipated in the earlier debate: first by discovering Denisovans, whose fossil record currently remains unrecognized, and second by revealing the multiplicity of admixture events, which include at least one that cannot be detected in present-day DNA.

## Populating Europe

Europe was first populated by modern humans around 45,000 ya, but (except for some southern areas) was depopulated during the glacial maximum that occurred 25,000 ya and subsequently repopulated as the climate improved, with farming beginning ~8000 ya during the Neolithic transition (Box 3). Decades of debate have been dedicated to understanding the origin of agriculture in Europe, focusing particularly on whether it spread from its place of origin in the Near East by demic diffusion (movement of farmers) or by acculturation of the indigenous hunter-gatherers (movement of ideas). Geneticists first attempted to answer this question by sampling modern populations from Europe and the Near East and then comparing the genetic diversity of classical protein markers (Box 1) between the two regions.

In 1978, the cover of *Science* magazine featured an image by Cavalli-Sforza and colleagues showing maps of Europe constructed with ten loci using multivariate techniques to reveal clines [23], which they interpreted as agreement with the demic diffusion model. Many genetic studies followed, investigating a variety of loci including mitochondrial DNA and the Y-chromosome. Some of these studies were uninformative, some were interpreted as supporting acculturation [24, 25] and others as favoring demic diffusion [26], with perhaps a balance supporting the latter [27].

Recent aDNA studies reveal, however, that populating Europe has been a much more complex process, and that the Neolithic transition (Box 3) was not even the event that most influenced the present-day genetic landscape.

**Fig. 1:**
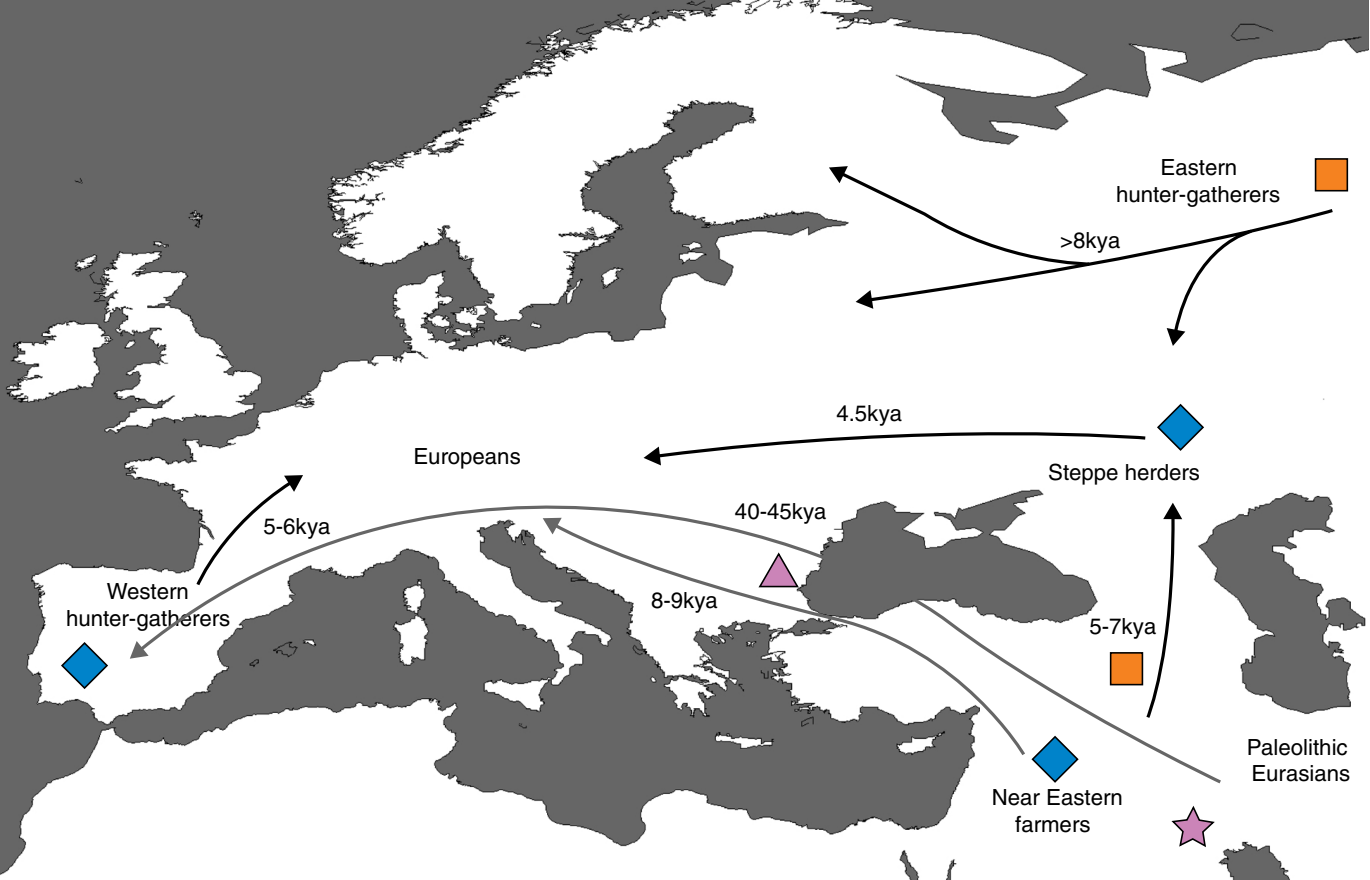
Populating Europe. aDNA research shows that present-day Europeans are the result of a mixture of different ancient populations (*blue diamonds*): (1) West hunter-gatherers who had inhabited Europe since Paleolithic times; (2) Early European farmers, who descended from Near Eastern farmers and entered Europe during the Early Neolithic; and (3) Steppe herders, who arrived in Europe during the Bronze Age. The steppe herders themselves were a mixture of eastern Eurasian hunter-gatherers (Eastern hunter-gatherers) and Near Easterners (*orange squares*). Additionally, Europeans have ~2 % archaic ancestry from mixture with Neanderthals that arose ~50,000–60,000 ya, probably somewhere in the Near East (*purple star*). There is also evidence that admixture with Neanderthals occurred again in Europe (*purple triangle*), as evident from the DNA of a 37,000–42,000-year-old human from Romania. However, this population appears not to have contributed detectably to later humans in Europe. *Grey arrows* represent the model for populating Europe inferred from modern DNA analysis. aDNA research refined this model by adding several additional layers of information, including multiple migrations and mixtures leading to present-day Europeans (*black arrows*)

The first aDNA complete genome sequence from Europe came from the Tyrolean Iceman; a 5300-year-old (Late Neolithic or ‘Copper Age’) natural mummy discovered in 1991 in the Ötztal Alps. Surprisingly, the Iceman had more genetic affinity to present-day Sardinians than to the present-day populations inhabiting the region where he probably lived [28], showing that major demographic changes have occurred in Europe after the Neolithic era. A more substantial revision of the demic diffusion model was introduced when several 7000–8000-year-old individuals from Western Europe [29] and a 24,000-year-old individual from Siberia [30] were sequenced. Analysis showed that at least three different ancient populations contributed to the genetics of present-day Europeans: (1) West European hunter-gatherers, (2) ancient north Eurasians related to Upper Paleolithic Siberians, and (3) early European farmers, who were mainly of Near Eastern origin [29]. The contributions of these three populations to modern European ancestry were not necessarily direct, and the demic diffusion model was further refined by analyzing 69 additional Europeans who lived between 3000 and 8000 ya (Fig. 1). The refined model shows that the arrival of the first farmers during the Early Neolithic from the Near East was followed by a massive migration from the Eurasian Steppe ~4500 ya involving people from the Yamnaya culture [31]. Controversially, these people were suggested to have also brought Indo-European languages into Europe [31]. The Yamnaya population distantly shares ancestry with the ancient Siberians; it is probably one of the sources of the Ancient north Eurasian ancestry previously identified among the three ancient populations that contributed to present-day Europeans [32]. It is worth noting here that the arrival of the Ancient north Eurasian ancestry in Europe through a surrogate population could not have been identified without analyzing the Yamnaya population: a reminder that even the interpretation of partial aDNA findings is vulnerable to the pitfalls of the parsimonious model. The genetic impact of the Yamnaya migration is strikingly illustrated by the transition in European Y-chromosomal haplogroups from a predominance of G2a beforehand to R1a and R1b afterwards [31], an impact that is, retrospectively, detectable in present-day DNA [33, 34].

In summary, aDNA findings have provided conclusive evidence for the movement of farmers at the beginning of the Neolithic transition, but also for the incorporation of the hunter-gatherer gene pool, and therefore support what might be called a ‘leaky demic diffusion’ model. In this respect, the new findings merge the previous ideas. But in demonstrating the large genetic contribution of the Yamnaya during the Bronze Age, they again reveal major events that were not anticipated in the earlier genetic debate.

## Origin of Native Americans

Although it has long been accepted that Native Americans’ ancestors migrated from Asia via Beringia (present-day Alaska) to occupy the Americas, much uncertainty has surrounded both their origin within Asia and the number of migrations. Genetic analyses show that Native Americans are most closely related to northeast Asians, but with different skull morphology. In 1996, the finding of a 8340–9200-year-old male human skeleton along the Columbia River shoreline outside Kennewick, Washington State, USA, heightened the debate on the origin of Native Americans. Initial assessment of the skeleton suggested he was anatomically distinct from modern Native Americans and more closely related to circumpacific groups such as the Ainu and Polynesians. Kennewick man, as the skeleton came to be known, was recently sequenced and found to be genetically closer to modern Native Americans than to any other population worldwide, therefore showing continuity with Native North Americans over at least the past eight millennia, despite the difference in morphology [35].

Insights into the Asian origin of the Native Americans came from a genome sequence of a 24,000-year-old boy found in Mal’ta in south-central Siberia [30]. The Mal’ta boy genome showed that Upper Paleolithic West Eurasians had a more north-easterly distribution and were genetically related to modern-day Native Americans, contributing significantly to their ancestry. This finding provided an explanation for some of the western Eurasian genetic signatures in present-day Native Americans, previously thought to be from post-Columbian admixture [30]. Modern East Asians appear to have replaced this ancient Eurasian population and hence have obscured the origin of the Native Americans. Additional insights into the origin of Native Americans came from the genome sequence of a ~12,500-year-old male infant (Anzick-1) recovered from the Anzick burial site associated with the Clovis culture in North America. Anzick-1 belonged to a meta-population from which many contemporary Native Americans are descended, and is closely related to all indigenous American populations. The ancient meta-population appears to have been related to Upper Paleolithic Asians, who probably reached the Americas a few thousand years before Clovis [36].

Controversy about the origin of Native Americans was recently sparked once more when analysis of present-day DNA from some Amazonians showed a small proportion of ancestry more closely related to indigenous Australians, Papuans and Andaman Islanders than to any present-day Eurasians or Native Americans, suggesting to the authors that there were two founding populations in the Americas [37]. However, another study using ancient and modern DNA reaffirmed the single-migration model for all Native Americans, detecting the same signal of gene flow from populations related to East Asians and, indirectly, to Australo-Melanesians but interpreting it as a later event. Native Americans appear to have diverged from Siberian ancestors ~20,000 ya, with another diversification occurring ~13,000 ya in the Americas leading to ‘northern’ and ‘southern’ Native American branches [38].

The Paleo-Eskimo cultures that settled in Greenland appear to have also originated from a migration from Siberia, but more recently (~5500 ya) and independently of the early migration that gave rise to the majority of Native Americans. Subsequent migration of the ancestors of the Inuit is evident from the genome sequence of ~4000-year-old Saqqaqman [39].

In this example, the aDNA data support and refine the existing models of the early peopling of the Americas, notably by informing on the different independent migrations and by simplifying the interpretation of the morphological differences between the first Americans and later Native Americans, showing these differences to be part of the same genetic continuum.

## Natural selection and introgression

Modern humans have come to inhabit an impressive diversity of ecological niches, many of which required local adaptation for survival. Several genetic signatures of adaptations have been identified by searching for alleles that are at high frequency in specific modern populations relative to other populations. For example, by studying genes that show population-specific allele-frequency differences between Tibetans and Han Chinese, a signal of positive natural selection was detected in *EPAS1*, a transcription factor involved in the response to hypoxia, which most probably helps the Tibetans to live at high altitudes [40]. Numerous other candidate genes that are under selective pressure related to immunity or subsistence have been identified in diverse populations. Nevertheless, estimation of the origin of the advantageous alleles or of the timing of the selection processes remained highly model-dependent until the use of aDNA. For example, the very unusual haplotype structure of the EPAS1 advantageous allele can now best be explained by introgression of DNA from Denisovans [41]. This conclusion is surprising because modern human ancestors and archaic hominins evolved separately for 550,000–765,000 years [14]; therefore, admixture between the two species is expected to have introduced alleles that reduced humans’ fitness. Indeed, strong purifying selection appears to have acted on the genome of modern humans to purge harmful archaic alleles. For example, genes that are highly expressed in testes have reduced Neanderthal ancestry [42]. On the other hand, archaic admixture appears to have also introduced a few beneficial alleles, such as *EPAS1*. Other examples include Neanderthal alleles that are enriched in genes affecting keratin filaments, which make up most of the outer layer of human skin and produce hair, suggesting that Neanderthal alleles may have helped modern humans to adapt to non-African cold environments [42]. The same may be true for Denisovan alleles, but the introgressed fragments remain to be identified.

Adaptation to non-African environments was also believed to be the cause of human variation in skin color. It was thought that the light skin of Europeans was a Paleolithic adaptation to facilitate vitamin D production in reduced sunlight regions [43]. Consistent with this hypothesis, aDNA analyses show that Scandinavian hunter-gatherers and Early European farmers indeed carried derived alleles contributing to light skin [44]. However, western hunter-gatherers of central and southern European populations survived in Palaeolithic Europe with dark skin pigmentation [44, 45]; thus, light skin has not been an essential adaptation for survival in this environment, and perhaps has resulted instead from sexual selection.

**Fig. 2:**
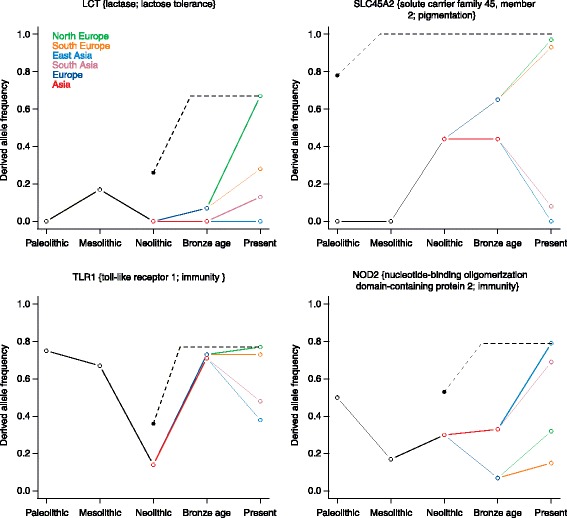
Recent positive selection in Europe and Asia. Change over time in the allele frequency of variants involved in adult lactose tolerance (rs4988235 for *LCT*), skin pigmentation (rs1426654 for *SLC24A5*) and immunity (rs4833095 for *TLR1* and rs9302752 for *NOD2*) observed from aDNA (*colored solid lines*). Allele frequencies in ancient populations are replotted from Allentoft et al. [32]. The *black dotted lines* represent predictions from theoretical models of selection based only on present-day population information. We assume that the initial frequency of the derived alleles in Eurasians in the past was similar to that in present-day Near Easterners. Increase in the frequency of these alleles was thought to have started in Paleolithic times (*SLC24A5*) or in Neolithic times (*TLR1*, NOD2 and *LCT*)

The warming after the last glacial period, followed by the Neolithic transition and the adoption of agriculture, introduced major changes in the lifestyle and diet of human populations. These events are proposed to have triggered new waves of selection that helped humans to adapt to the resulting social and environmental changes. In particular, selection on immune genes was believed to have been increased by the spread of diseases after the Neolithic due to dense settlements and proximity to domesticated animals. aDNA from Mesolithic Europeans shows, however, that adaptive variants associated with pathogen resistance in modern populations were already present in hunter-gatherers before the advent of agriculture [45]. In fact, only a limited number of strong selective sweeps associated with diet and pigmentation can be associated with agriculture in Europe [44]. Even lactose tolerance, assumed to be associated with pastoralism, appears to have been absent in early European farmers, being found in only 10 % of Bronze Age Europeans and increasing dramatically in just the past 3000 years [32, 44] (Fig. 2).

aDNA findings are thus now beginning to transform our understanding of recent positive selection in humans, both by introducing new mechanisms such as adaptive introgression, and by showing that our estimates of the timing of selective sweeps derived from models using present-day populations were unreliable, with the consequence that some widely held hypotheses about the selective forces were also unreliable.

## Conclusions

Findings from aDNA research are currently transforming our understanding of human history at an ever-increasing pace. When evolution was parsimonious, aDNA may support the prevailing model, as with the initial peopling of the Americas; but more often, evolution was *not* parsimonious, and aDNA reveals a much richer history, as in the other examples considered here. In either situation, human evolutionary genetics is moving to a paradigm where we first look for evidence from aDNA and interpret present-day genetic variation in its light.

What are the limits to how far this can go? Very ancient samples more than 100,000 years old and some geographical regions of great interest, such as the Near East and Africa, remain challenging for aDNA research. Both time and poor DNA preservation in hot wet climates may impose insurmountable limits to resolving many questions related to the origin and genetic diversity of our species. Identifying favorable locations within these regions [46], or relevant relict populations and migrant individuals, offers some ways around such limitations. Improvements in aDNA extraction and library construction will push the limits, but sequences below 25 base pairs in length often do not map uniquely to the human genome, and so provide little useful information. There is room for methodological improvements in repair and perhaps reconstruction of ancient molecules within the fossils.

In the near future, we look forward to insights into human history ranging from hundreds of thousands of years ago to the past few centuries. Can we obtain nuclear sequences from *Homo heidelbergensis* (‘Sima de los Huesos’) or any sequence data from *Homo floresiensis* or *Homo erectus*? Who were the sources of the non-Neanderthal, non-Denisovan archaic admixture already detected? What did Denisovans look like? What were the number, timing and routes of the major expansion(s) of fully modern humans out of Africa? What was the full richness of subsequent human population history and adaptation throughout the world, including episodes that have left no traces in present-day populations? We expect our understanding to be transformed again in these and unforeseen directions, perhaps even before this review is published.

## Box 1 The evolution of genetic studies: from ‘markers’ to whole-genome sequences

Over the past 100 years, the datasets and mathematical methodologies used in population genetics have changed enormously, providing an ever better understanding of human genetic diversity over time and space. In 1954, Arthur Mourant published his ground-breaking book “*The distribution of the human blood groups*” [47], probably the first full anthropological work to use a genetic perspective, showing that detectable genetic differences exist among different human populations. Blood groups and protein types constitute what are now known as ‘classical markers’ and were used to compare human populations for several decades, preceding the DNA-based datasets utilized today.

The development of the polymerase chain reaction (PCR) in the 1980s introduced the use of molecular markers to population genetics and allowed, for the first time, the study of evolutionary distances between alleles at a locus. This methodological progress, along with theoretical advances such as identity by descent developed by Gustave Malécot in 1939 [48] and coalescent theory developed by John Kingman in 1982 [49], provided an unprecedented understanding of the genetic relationships among human populations, as well as their relatedness and divergence from other species.

The first widely used molecular markers were variants of the mitochondrial DNA (mtDNA) and the non-recombining region of the Y-chromosome (NRY). mtDNA is inherited maternally and transmitted from a mother to her children, while the NRY is inherited paternally passing down from father to son. These uniparental markers are transmitted from one generation to the next intact (apart from new mutations) and have known mutation rates, allowing straightforward construction of phylogenies and inference of some aspects of population relationships. Uniparental loci are, however, sex-specific and experience strong drift, providing a limited view of the complex human history. For example, Neanderthal mtDNA analysis shows no evidence of admixture with modern humans [50], although admixture has occurred and is detectable when the whole genome is considered.

The study of genome-wide markers was initiated using microsatellites (short tandem repeats, STRs) but was simplified by the development of single nucleotide polymorphism (SNP) arrays. The effective population size of autosomal variants is expected to be four times that of mtDNA and NRY, making autosomal variants less prone to drift and providing insight further back into human history. Nevertheless, inferences from SNP arrays are limited by ascertainment biases arising from their design, which generally incorporated SNPs that were discovered in a few populations and were inadequate to capture global genetic diversity.

The development of next-generation sequencing (NGS) resolved many of the limitations of the previous methodologies by generating gigabases of sequence data from the whole genome, reducing ascertainment biases while increasing the power to detect evolutionary processes. NGS produces large numbers of short sequencing reads. This feature is particularly useful for ancient DNA analysis and has allowed the sequencing of genomes that are tens of thousands of years old, making the direct study of the evolutionary changes over time and space possible. NGS is thus currently revolutionizing the field of population genetics.

## Box 2 D-statistics

Patterson’s D-statistic is a comparative measure of allele sharing between two populations and an outgroup. It can be used as a formal test for admixture and can provide information about the direction of gene flow. It was first introduced by Green et al. [9] to show that Eurasian populations share more derived alleles (i.e., alleles different from the ancestral (chimpanzee) allele) with Neanderthals than do Africans, a signal interpreted as evidence for archaic introgression in modern humans. The D-statistic assumes that populations fall within a phylogeny where the relationships between populations are known: for example, Green et al. used *D(Human1, Human2, Neanderthal, Chimpanzee)* and looked at the derived alleles in Neanderthal. The ancestral allele is defined by the Chimpanzee sequence and is labeled *A*, the derived allele is labeled *B*. Two possible patterns of SNPs can then be observed and counted: ‘ABBA’ or ‘BABA’. If *Human1* and *Human2* shared the same history in their relation to Neanderthal, they will not differ in their derived allele frequencies (ABBA and BABA will occur with equal frequencies in the two human populations), and *D* will not differ significantly from zero. Gene flow from Neanderthal to one of the human populations will lead to an increase in the derived alleles that have occurred on the Neanderthal branch in that human population and D will deviate from zero. Green et al. performed the following test: *D(African, Eurasian, Neanderthal, Chimpanzee)* and found *D* was always positive from a significant excess of ABBA sites over BABA sites. The test has subsequently become widely used in many different ways.

## Box 3 The Neolithic transition

The Neolithic transition or revolution refers to the change in lifestyle of humans from hunting-gathering to agriculture, through domestication of plants and animals, which led to the development of permanent settlements, towns, cities, trade and eventually the rise of civilization. Neolithic transitions occurred independently in several parts of the world; the one that transformed Europe started in the Near East around 10,000 years ago and spread through Europe over the next few millennia. This cultural transition had considerable consequences for human genetic variation by stimulating growth in population size, and triggering multiple expansions and mixtures, as well as adaptation to certain diets and diseases.

## Abbreviations

aDNA: Ancient DNA
kb: Kilobabases
kya: thousands of years ago
Mb: Megabases
mtDNA: Mitochondrial DNA
NGS: next-generation sequencing
NRY: non-recombining region of the Y-chromosome
SNP: single nucleotide polymorphism
ya: years ago
